# Intratumoral radiofrequency hyperthermia-enhanced direct chemotherapy of pancreatic cancer

**DOI:** 10.18632/oncotarget.12295

**Published:** 2016-09-27

**Authors:** Zhibin Bai, Yaoping Shi, Jianfeng Wang, Longhua Qiu, Eric J. Monroe, Gaojun Teng, Feng Zhang, Xiaoming Yang

**Affiliations:** ^1^ Image-Guided Biomolecular Intervention Research, Section of Interventional Radiology, Department of Radiology, University of Washington School of Medicine, Seattle, WA, USA; ^2^ Department of Radiology, Zhongda Hospital, Southeastern University, Nanjing, China; ^3^ Department of Interventional Oncology, Renji Hospital, School of Medicine, Shanghai Jiaotong University, Shanghai, China

**Keywords:** radiofrequency, hyperthermia, chemotherapy, pancreatic cancer, local

## Abstract

**Purpose:**

To investigate the technical feasibility of using ultrasound-guided intratumoral radiofrequency hyperthermia (RFH) to enhance local chemotherapy of rat orthotopic pancreatic cancers.

**Materials and Methods:**

Orthotopic pancreatic cancer masses were established by inoculating luciferase/mCherry labeled-pancreatic cancer cells into the pancreatic tails of Lewis model rats via a laparotomy approach. Twenty-four rats with pancreatic cancer and 24 mice with subcutaneous pancreatic cancer xenografts in four study groups (*n* = 6/group) received various treatments: i) combination therapy of intratumoral MR imaging-heating-guidewire-mediated RFH (42^o^C) plus local chemotherapy (gemcitabine); ii) intratumoral chemotherapy alone; iii) RFH alone; and (iv)phosphate-buffered saline (PBS). Transcutaneous ultrasound imaging was used to guide the treatment and subsequently follow changes in tumor sizes. Bioluminescence optical imaging was performed to follow photon signal changes. Sonographic and optical findings were correlated with histology at 14 days.

**Results:**

Optical imaging demonstrated a significantly decreased bioluminescence signal in mice with combination therapy group, compared with the other control groups (0.51±0.18 *VS* 1.6±0.4 *VS* 3.18±0.9 *VS* 3.5±0.96, *p* < 0.05). Ultrasound imaging showed the smallest tumor volumes of both mice and rat group with the combination therapy, compared with other control groups (0.62±0.16 *VS* 1.25±0.19 *VS* 2.28±0.25 *VS* 2.64±0.26, *p* < 0.05) and (0.75±0.18 *VS* 1.31±0.30 VS 1.61±0.28 *VS* 1.72±0.28, *p* < 0.05). Both imaging findings were confirmed by histologic correlation.

**Conclusion:**

Intratumoral RFH can augment the chemotherapeutic effect in an orthotopic pancreatic cancer model.

## INTRODUCTION

Pancreatic cancer is one of the most lethal abdominal malignancies and is the fourth leading cause of cancer-related death in Western nations. At the time of diagnosis, more than 80% of patients present with either an advanced unresectable tumor or metastatic disease [1, 2]. In cases of unresectable disease, the median survival is around 6 months [3-6]. Therefore, it is essential to develop novel and effective therapies for patients with unresectable pancreatic cancer [4-6].

Gemcitabine-based chemotherapy has been accepted as the first line treatment for patients with unresectable and locally advanced pancreatic cancer, but offers only a limited survival benefit of 3 months [7-9]. The unsatisfactory therapeutic effect of systemic chemotherapy is associated with the difficulty of delivering sufficient drugs to highly desmoplastic pancreatic cancer tissues [8]. Radiofrequency (RF)-mediated ablative thermal energy has been increasingly used to treat solid parenchymal tumors. RF ablation of unresectable pancreatic tumors has been reported [3, 7, 9-11], but carries a high risk of pancreatitis and severe thermal injuries to critical structures adjacent to the pancreatic tumors, such as duodenum, portal vein or common bile duct [3, 12-14]. Some serious complications require operative intervention and therefore surgery despite the presence of unresectable tumor [3].

Recent studies have confirmed that subablative hyperthermia around 42^o^C can significantly improve chemotherapeutic effect on different cancers via increasing the sensitivity of cancer cells to chemotherapeutic drugs and reversing chemo-resistance [1, 15, 16]. However, in clinical practice, such subablative hyperthermia for treating malignancies has been generated by either whole body hyperthermia or external hyperthermia around the body [1, 17, 18]. In the last decade, we have developed a radiofrequency heating guidewire, which functions as an intraluminal thermal energy source for locally enhancing gene/chemotherapies [16, 19, 20]. The purpose of this study was to investigate the technical feasibility of using ultrasound-guided intratumoral radiofrequency hyperthermia (RFH) to enhance local chemotherapy of rat orthotopic pancreatic cancers.

## MATERIALS AND METHODS

### Study design

We divided the study into three phases: (1) in-vitro experiments to establish “proof-of-principle” of the new concept, RFH-enhanced chemotherapy of pancreatic cancer, using rat pancreatic cancer cells (DSL-6A/C1); (2) in-vivo confirmation of the new concept on mice models with molecular imaging-detectable subcutaneous pancreatic cancer xenografts; and (3) preclinical feasibility validation of the new technique using a rat models with orthotopic pancreatic cancers.

### *In vitro* experiments

#### Cell culture and RFH-enhanced chemotherapy

Rat pancreatic cancer cells (DSL-6A/C1) were transfected with luciferase (Luc)/red fluorescence protein (RFP) /lentivirus gene to create Luc/RFP-positive cells according to the protocol provided by the manufacturer (GeneCopoeia Inc., Rockville, MD). Luc/RFP-positive cells were sorted out using fluorescence-activated cell sorting technique (Aria II, Becton Dickinson, Franklin Lakes, NJ). Cells were then seeded in four-chamber cell culture slides (Nalge Nunc International, Rochester, NY) and maintained in Waymouth's MB 752/1 medium (Gibco, Grand Island, NY) supplemented with 10% fetal bovine serum (FBS) (Gibco). RF hyperthermia was performed as described in the literature [20]. Cells in different groups were treated by (a) Gemcitabine (5.0 μM) plus 30-min RFH at approximately 42^o^C; (b) Gemcitabine alone; (c) 30-min RFH alone; and (d) no treatment to serve as a control. We used the 50-percentage inhibitory concentration (IC50) dose of gemcitabine for cell treatment, which was decided by CellTiter 96 Aqueous One Solution Cell Proliferation-assay (Promega Corporation, Madison, WI).

#### Cell viability assay

Cells proliferation was evaluated by CellTiter 96 Aqueous One Solution Cell Proliferation-assay 48 hours after treatments. Relative cell proliferations of different cell groups were evaluated using the equation of A_treated_-A_blank_ / A_control_-A_blank_, where A is absorbance. Cells on slides were subsequently washed twice with phosphate-buffered saline (PBS), fixed in 4% paraformaldehyde, counterstained with 4’,6-diamidino-2-phenylindole (DAPI, Vector Laboratories, Burlingame, CA), and then imaged with a confocal microscopy. All experiments for each of cell groups were repeated six times.

### *In vivo* experiments

The animal protocol was approved by our Institutional Animal Care and Use Committee. The animals were anesthetized with 1%-3% isoflurane (Piramal Healthcare, Andhra Pradesh, India) in 100% oxygen.

#### Mouse model for proof of principle

Mouse models with pancreatic cancer xenografts were created on 24 nu/nu mice aged 4-6 weeks (Charles River Laboratories, Wilmington, MA) by subcutaneously inoculating 5×10^6^-1×10^7^ Luc/RFP-positive DSL-6A/C1 cells in 100 μl Matrigel into the left back of each mouse. Once the size of tumor reached 5-10mm in diameter, we began the experimental procedures.

When the tumors grew to around 5-10 mm in diameter, six mice in each of four groups were treated by (a) intratumoral injections of gemcitabine (20-mg/kg for mice) in 100 μL PBS, followed by RFH at approximately 42°C for 30 mins; (b) intratumoral injection of gemcitabine alone; (c) 30-min RFH alone; and (d) injection of 100 μL PBS to serve as a control. RF hyperthermia was performed as described in the literature [16].

#### Pre-clinical validation of the new technique feasibility using rat models

Rat models with orthotopic pancreatic cancer xenografts were created on Lewis rats weighted 150-200g (Harlan Laboratories, Livermore, CA). A total of 5×10^6^-1×10^7^ DSL-6A/C1 cells were first subcutaneously injected into right flanks of 6 donor Lewis rats. The subcutaneous tumors were excised under aseptic conditions once they grew to 10-15 mm in the largest diameter. The tumors were harvested and minced by a scalpel into small fragments of 1 mm^3^ in size.

For each of the 24 recipient Lewis rats, the abdomen was opened through a median incision and the spleen pancreas and pancreatic tail were gently exposed. A tumor bed was prepared in the pancreatic parenchyma with a microscissor (RS-5610 VANNAS; Roboz, Rockville, MD). Five tumor fragments were placed into pancreatic tissue bed, completely wrapping the tumor tissue by pancreatic parenchyma. The pancreas was relocated into the abdominal cavity and the median incision was closed in two layers with 5-0 absorbable sutures.

When the orthotopic pancreatic tumors had grown to 5-10 mm in diameter, six rats in each of four groups were treated by (a) intratumoral injections of gemcitabine (100-mg/kg for rats) in 100 μL PBS, followed by RFH at approximately 42°C for 30 mins; (b) intratumoral injection of gemcitabine alone; (c) 30-min RFH alone; and (d) injection of 100 μL PBS to serve as a control. Gemcitabine in 100 μL PBS was directly injected into the tumor mass through the percutaneous puncture approach under ultrasound guidance. Immediately after the drug delivery, RF hyperthermia was delivered by inserting a 0.022-inch MR imaging heating guidewire into tumor mass via the gemcitabine injection needle, with its heating spot located at the center of tumor mass. A fiberoptic temperature probe was placed in the tumor margin parallel to MR imaging heating guidewire to monitor the temperature under ultrasound guidance. The temperature was kept at around 42°C for 30 minutes by adjusting the RF output power at 10 watts.

#### Following tumor growth with bioluminescent (BL) optical imaging and ultrasound imaging

Optical imaging was conducted on a Bruker In-Vivo Xtreme Imaging Systems (Bruker Corp., Billerica, MA). For mice, each animal was imaged at day 0 before treatment and at days 7 and 14 after the treatment. Animals were anesthetized with 1%-3% isofluorane in 100% oxygen. Bioluminescent images were acquired twenty minutes after intraperitoneal injection of Pierce D-Luciferin (150mg/Kg) (ThermoFisher Scientific, Pittsburgh, PA). Signal intensity was quantified using the Bruker MI software. Relative signal intensity (RSI) was calculated by using the equation: RSI = SI_Dn_/SI_D0_, where SI is signal intensity, Dn represents days after treatment, and D0 is the day before treatment.

Ultrasound imaging was performed to follow the tumor growth (Sonosite Inc, Bothel, WA) at day 0 before treatment and at days 7 and 14 after the treatment for both mice and rats. The axial (X) and longitudinal (Y) diameters of tumors, as well as tumor depths (Z) were measured on the ultrasound images. The volume of each tumor mass was calculated according to equation of volume= X*Y*Z*π/6. Data was expressed as relative tumor volume (RTV) by using the following equation: RTV = V_Dn_/V_D0_, where V is tumor volume, Dn represents days after treatment, and D0 is the day before treatment.

#### Histologic correlation/confirmation

Tumors were harvested 14 days after treatments and cryosectioned at 8-μm slices for apoptosis staining. Level of apoptosis was determined with a terminal deoxynucleotidyl transferase dUTP nick end labeling assay (TUNEL) using TACS XL Blue Label kit (Trivegen, Gaithersburg, MD). On one slide, six fields were randomly photographed using an Olympus DP72 digital camera. Apoptosis results were analyzed as the apoptotic index, defined as the number of apoptotic cells / total number of cells ×100%.

#### Statistical analysis

Statistical software SPSS 19.0 (SPSS, Chicago, Ill) was used for all data analyses. A non-parametric Mann-Whitney U test was used to compare (i) relative proliferation rates among different cell groups; (ii) relative optical signal intensities as well as (iii) relative tumor volumes at different time points among various animal groups. P value of less than 0.05 was considered significant.

## RESULTS

### *In vitro* confirmation: RFH-enhanced chemotherapeutic effect on pancreatic cancer cells

**Figure 1:**
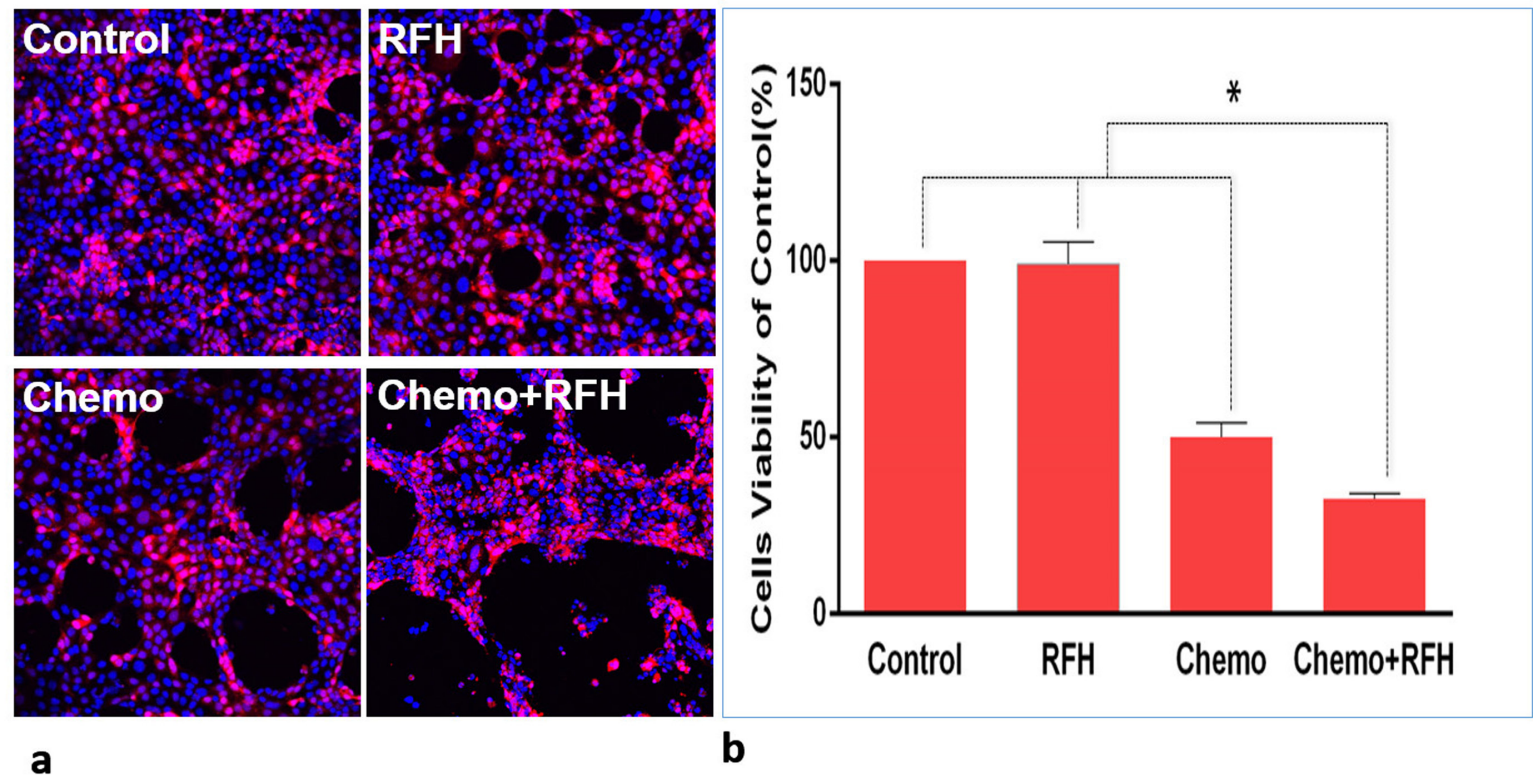
*In vitro* experiments using Luciferase-labelled pancreatic cancer cells **a.** Confocal microscopy shows the lowest numbers of survival cells in the combination treatment (Chemo+RFH). **b.** MTS assay demonstrating the lowest cell viability in the group receiving combination treatment (Chemo+RFH), compared with those of control groups (*=*p* < 0.05).

Confocal microscopy of cells in each treated group showed that much less cells survived after the treatment in combination therapy than three other groups (Figure 1a), which was consistent with the results of proliferation assay. CellTiter 96 Aqueous One Solution Cell Proliferation-assay demonstrated the lowest cell viability of Chemotherapy plus RFH, compared to those of groups with chemotherapy-only, RFH-only, and PBS (32.71±1.34% *VS* 50.22±3.96% *VS* 99.23±6.09% *VS* 100%, *p* < 0.05) (Figure 1b).

### *In vivo* confirmation: RFH-enhanced chemotherapy on mouse subcutaneous pancreatic cancers

**Figure 2:**
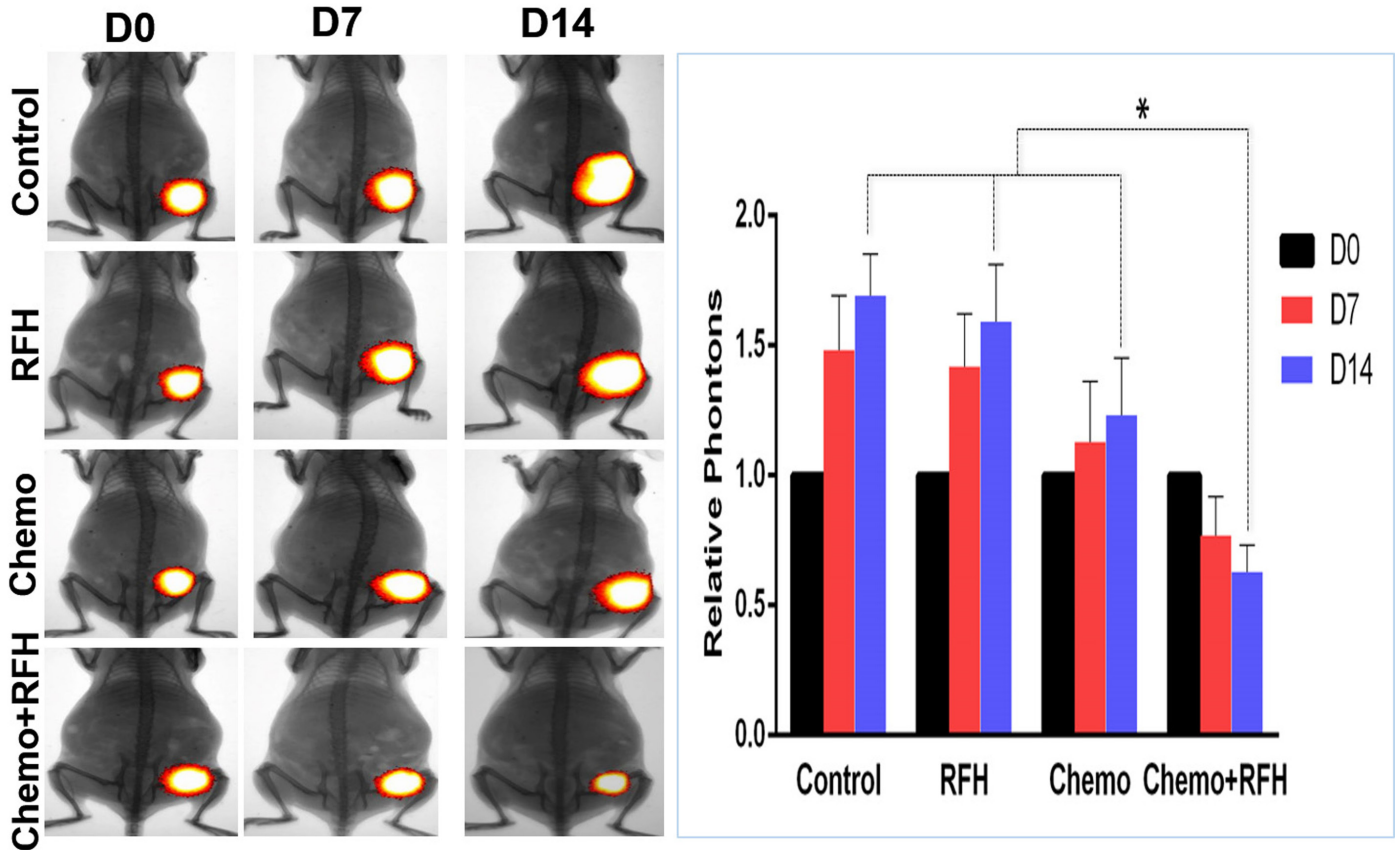
*In vivo* experiments on mouse models with subcutaneous pancreatic cancers (Left) Optical/x-ray imaging is used to follow up the tumor growth at days 0, 7 and 14 after treatments, showing significantly decreased florescent signals (yellow-red color) in the group with combination therapy (Chemo +RFH), compared to those of other three control groups (Right, * *p* < 0.05).

**Figure 3:**
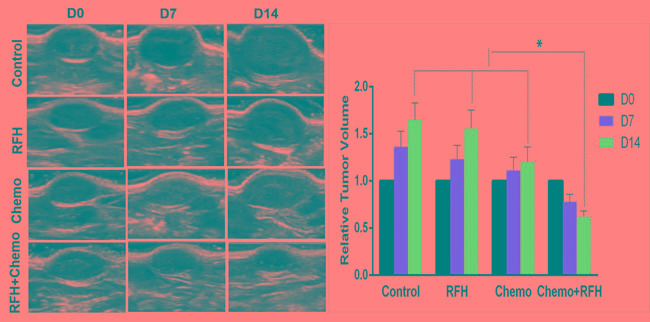
Ultrasound imaging of mice with subcutaneous pancreatic cancers (Left) Follow tumor growth at days 0, 7 and 14 after treatments, showing a significant decrease of average tumor volume in the group with combination therapy (Chemo +RFH), compared to the three control groups (Right, * *p* < 0.05).

**Figure 4:**
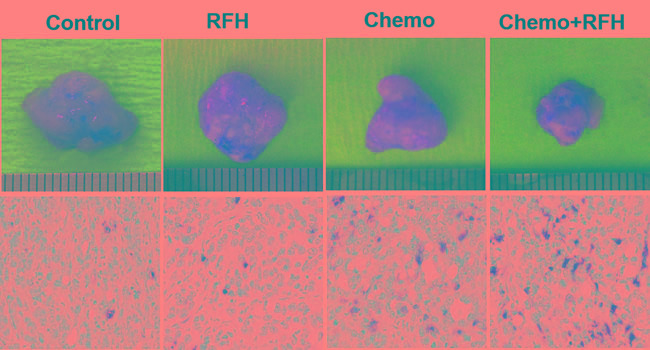
Histology of mice with subcutaneous pancreatic cancers (Upper row) Representative tumors harvested from four different mouse groups, showing the smallest tumor size in combination therapy group (Chemo+RFH) compared with other three treatments. (Lower row) Apoptosis analysis using TUNEL staining (20X magnification) demonstrates more apoptotic cells (brown dots) in the combination therapy group (Chemo+RFH) than those of three control groups.

All mice survived the procedures without any major complications. Optical imaging showed a significant decrease of relative photon signal intensities for the combination therapy group as compared to those of chemotherapy-only group, RFH-only group, and control group (0.63±0.10 *VS* 1.23±0.22 *VS* 1.59±0.22 *VS* 1.69±0.16, *p* < 0.05) (Figure 2). Ultrasound imaging of tumors showed the smallest relative tumor volumes in combination therapy group compared with control groups (0.62±0.06 *VS* 1.20±0.16 *VS* 1.56±0.19 *VS* 1.65±0.18, *p* < 0.05) (Figure 3). Histology of tumor size examination and apoptosis analysis showed the smallest tumor volume in the combination group, compared with other three groups, which were well correlated with imaging findings (Figure 4).

### *In vivo* validation: RFH-enhanced chemotherapy on rat orthotopic pancreatic cancers

**Figure 5:**
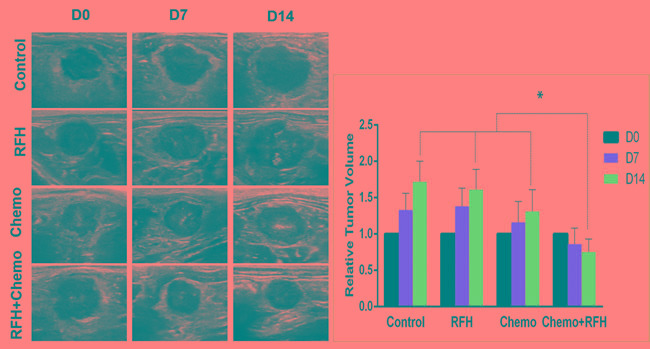
*In vivo* experiments with rat orthotopic pancreatic cancers (Left) Representative ultrasound imaging follow-up in four different animal groups with various treatments, showing the smallest tumor size (T) for the combination treatment (RFH+Chemo). (Right) quantified analysis confirmed a significant decreased relative tumor volume in the combination therapy group at day 14 compared with those of other three control animal groups (Right, *p < 0.05).

**Figure 6:**
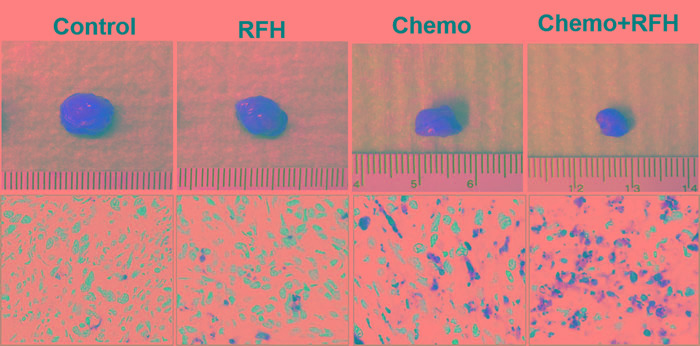
*In vivo* experiments on rat models with orthotopic pancreatic cancers (Upper row) Representative tumors harvested from four different animal groups, showing the smallest tumor size in combination therapy group (Chemo+RFH) compared with other three treatments. (Lower row) Apoptosis analysis using TUNEL staining (20X magnification) further confirms more apoptotic cells (brown dots) in the combination therapy group (Chemo+RFH) than those of three control groups.

Chemotherapy plus RFH significantly inhibited the growth of tumors in rat orthotopic pancreatic cancer xenografts (Figure 5). Fourteen days after the treatment, the average relative tumor volume was the smallest in the chemotherapy plus RFH group, as compared with the relative tumor volume of chemotherapy-only group, RFH-only group, and the control group (0.75±0.18 *VS* 1.31±0.30 *VS* 1.61±0.28 *VS* 1.72±0.28, *p* < 0.05). The significant tumor volume decrease in the combination therapy group was well consistent with the results of apoptosis analysis of tumors (Figure 6).

## DISCUSSION

In our study, we initially carried out *in vitro* experiments to establish the “proof-of-principle” of the concept, using RFH to enhance the killing effects of gemcitabine on pancreatic cancer cells. We then further confirmed the principle by *in vivo* experiments on mouse models with subcutaneous pancreatic cancers. Then, we validated the feasibility of this new technique on rat models with orthotopic pancreatic cancers. We found RFH significantly enhanced the direct intratumoral chemotherapy of pancreatic cancers, both decreasing the number of survived rat pancreatic cancer cells in the in-vitro experiments and decreasing tumor volumes in both the mouse and rat.

Single-agent gemcitabine is a standard therapeutic strategy for locally advanced and metastatic pancreatic cancer, slightly improving overall survival (OS) and clinical benefit compared with fluorouracil (FU) (5.6 *vs*. 4.4 months) [21]. However, the overall response rate of pancreatic cancers to gemcitabine remains low [2, 22]. In current clinical practice, chemotherapy for pancreatic malignancies is usually carried out via systemic administration, limiting the dose of chemotherapeutics to the pancreatic targets and often causing toxicities to other vital organs. Radiofrequency ablation (RFA) is a local ablative method that can destroy tumors by thermal coagulation and protein denaturation. RFA has been used successfully in the treatment of unresectable solid tumors in the liver, lung, and kidney [3, 13]. The delicate nature of the pancreatic parenchyma predisposes it to pancreatitis caused by RFA-mediated thermal damage. Furthermore, closely adjacent critical structures such as duodenum, portal vein and common bile duct are at risk of thermal injury. These limitations have been the main impeding factors in application of RFA to treat pancreatic cancer [12].

Studies reported that radiofrequency-induced mild hyperthermia can enhance cytotoxicity of many chemotherapeutics through mechanisms of increased drug uptake, increased chemo-sensitivity and decreased the chemoresistance [1, 15, 16]. However, the precise and effective delivery of hyperthermia solely to the target tumor mass remains a serious technological challenge, particularly for deep-seated tumors such as pancreatic malignancies. Under ultrasound imaging guidance, we placed the heating spot of a RF heating guidewire in the center of the pancreatic cancer mass for delivering hyperthermal energy to the tumor. Fiber optical temperature probe placement at the margin of the target tumor allowed precise control RFH within the tumor at a temperature around 42°C. Our study indicates the potential of taking the advantage of non-ablative hyperthermia to augment the chemotherapy while minimizing RFA-associated thermal compilations.

Development of novel, clinically meaningful therapeutic approaches for pancreatic cancer highly relies on the availability of preclinical animal models that can resemble the anatomic and pathophysiological features of the disease. Such animal models also offer high predictive value for evaluating any new diagnostic and therapeutic technologies [23-25]. Usually, *in vivo* pancreatic cancer animal models are created on immunodeficient mice [26, 27]. These models suffer from an inherently deficient interaction of implanted cancer cells with host immune system and do not permit evaluation of the treatment-associated complications to adjacent critical structures[28]. Through the current study, we have successfully created a rat model with orthotopic pancreatic cancer to serve as useful platform for exploring new diagnostic and therapeutic methods.

This study has several limitations. The optimal temperature for maximally enhanced chemotherapeutic effect remains to be characterized and further work in this regard is warranted. This work primarily focused on technical development and suffers from a lack of a systemic chemotherapy control group. We were not able to follow up the animals longer than 14 days, because the tumors in the control groups might grow too large in excess of the institutional-required limit of 10% body weight.

We concluded that intratumoral RFH can augment the chemotherapeutic effect in an orthotopic pancreatic cancer model. This development may open a new avenue for effective management of pancreatic malignancies by simultaneous integration of radiofrequency technology, interventional oncology, and direct intratumoral chemotherapy.
